# Dual-Labeled Small Peptides in Cancer Imaging and Fluorescence-Guided Surgery: Progress and Future Perspectives

**DOI:** 10.3390/ph18020143

**Published:** 2025-01-22

**Authors:** Paul Minges, Matthias Eder, Ann-Christin Eder

**Affiliations:** 1 Department of Nuclear Medicine, University Medical Center Freiburg, Faculty of Medicine, University of Freiburg, 79106 Freiburg, Germany; paul.minges@uniklinik-freiburg.de (P.M.); m.eder@dkfz.de (M.E.); 2 Department of Radiopharmaceutical Development, German Cancer Consortium (DKTK), Partner Site Freiburg, Freiburg, Germany and German Cancer Research Center, 69120 Heidelberg, Germany

**Keywords:** dual imaging, dual labeling, hybrid molecule, dual-modality imaging, peptidomimetics, small molecules, fluorescence labeling, isotope labeling, fluorescence-guided surgery

## Abstract

Dual-labeled compounds that combine radiolabeling and fluorescence labeling represent a significant advancement in precision oncology. Their clinical implementation enhances patient care and outcomes by leveraging the high sensitivity of radioimaging for tumor detection and taking advantage of fluorescence-based optical visualization for surgical guidance. Non-invasive radioimaging facilitates immediate identification of both primary tumors and metastases, while fluorescence imaging assists in decision-making during surgery by offering a spatial distinction between malignant and non-malignant tissue. These advancements hold promise for enhancing patient outcomes and personalization of cancer treatment. The development of dual-labeled molecular probes targeting various cancer biomarkers is crucial in addressing the heterogeneity inherent in cancer pathology and recent studies had already demonstrated the impact of dual-labeled compounds in surgical decision-making (NCT03699332, NCT03407781). This review focuses on the development and application of small dual-labeled peptides in the imaging and treatment of various cancer types. It summarizes the biomarkers targeted to date, tracing their development from initial discovery to the latest advancements in peptidomimetics. Through comprehensive analysis of recent preclinical and clinical studies, the review demonstrates the potential of these dual-labeled peptides to improve tumor detection, localization, and resection. Additionally, it highlights the evolving landscape of dual-modality imaging, emphasizing its critical role in advancing personalized and effective cancer therapy. This synthesis of current research underscores the promise of dual-labeled peptides in enhancing diagnostic accuracy and therapeutic outcomes in oncology.

## 1. Introduction

In the vast field of cancer treatment, radioactive isotopes have demonstrated high efficacy in both diagnosis and therapy. Tracer molecules, designed for targeted radionuclide delivery to tumors, play a pivotal role in this process. The emitted radiation is utilized for direct treatment or diagnosis by imaging techniques [1]. In general, radiopharmaceuticals comprise a targeting component, such as a nanoparticle, small molecule, peptide, nanobody, or antibody; a radionuclide-binding moiety, e.g., a chelator; and a linker region [2]. A prominent example of a radiopharmaceutical is [^177^Lu]Lu-PSMA-617, which contains a small peptide as a binding motif and is utilized in the treatment of PSMA-positive metastatic castration-resistant prostate cancer (mCRPC) [3]. Initially published in 2015 [4], it was subsequently approved by the Food and Drug Administration in 2022 [5]. However, the preoperative identification of primary tumors and metastases can be performed precisely with compounds such as PSMA-targeting diagnostic radiopharmaceuticals by PET/CT imaging [6]. Nonetheless, the accurate intraoperative localization and delineation of tumor margins and lymph node metastases remain a major challenge. For prostate carcinoma, the established workaround is a template-based removal of nonpathological lymph nodes with the drawback of increased morbidity. Additionally, there is an enhanced risk of residual tumorous structures [2,7].

**Figure 1:**
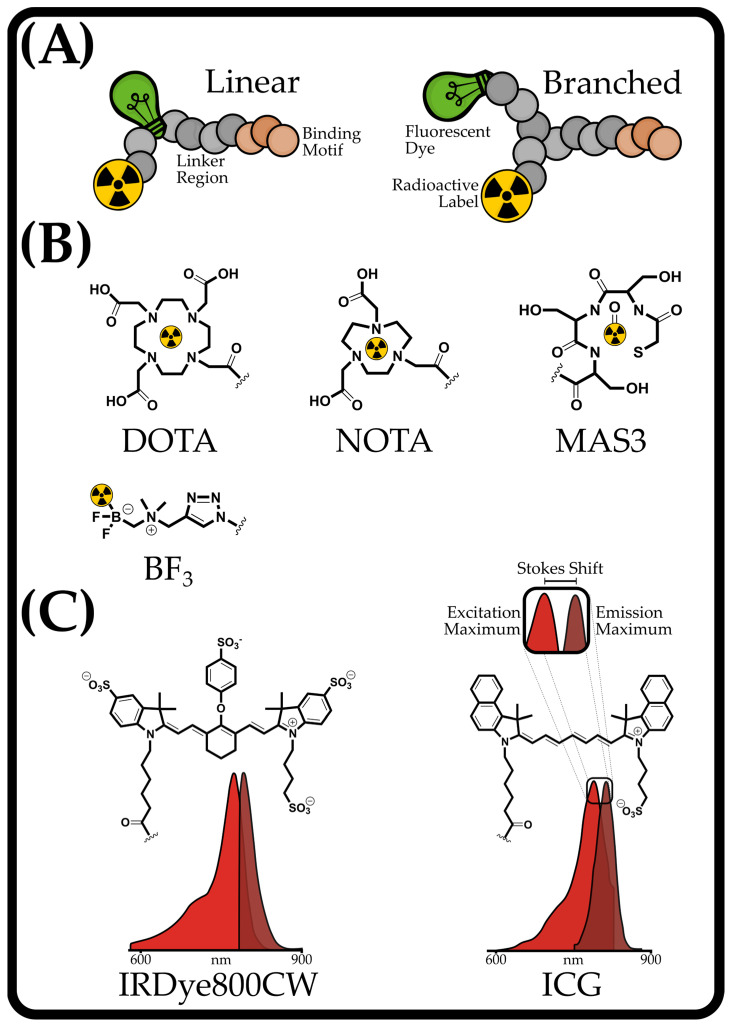
Illustration of the fundamental structure of dual-labeled small peptides, with the amino acids (aa) represented as circles. (**A**) outlines the two approaches for dual-labeled peptides: either the fluorophore (green light bulb) is incorporated linearly with the chelator (radioactive symbol) into the peptide (Linear) or the peptide has a branch, with both ends capable of labeling (Branched). The receptor binding motif (colored aa) is usually located at one end of the peptide, and the three components are connected via a linker (grey aa). (**B**) shows a selection of strategies for radiolabeling: DOTA (^68^Ga, ^111^In, ^177^Lu), NOTA (^68^Ga), MAS3 (^99m^Tc), and BF_3_ (^18^F). (**C**) presents two fluorescent dyes utilized in clinical settings, namely, IRDye800CW and ICG, showing their structures and excitation/emission spectra. Additionally, the Stokes shift is elaborated upon by examining the ICG spectra, which describes the difference in nanometers between the excitation and emission maximum.

Starting from this, about two decades ago, in 2005, an article was published reporting the first small peptide that introduced an additional fluorescent dye as a second label to an existing radioactive tracer to overcome the limitations of single-labeled compounds (general structure of dual-labeled small peptides shown in Figure 1A). As a result, a new variable was introduced, exerting influence on pharmacological behavior. On the one hand, the chemical structure determines the fluorescence properties, including the absorption/emission properties, the Stokes shift, and the quantum yield (Figure 1C). At the same time, it determines the pharmacodynamics of the compound through its molecular mass, net charge, and steric orientation [8]. The existing landscape of utilized fluorophores remains underdeveloped, thus offering numerous avenues for enhancement. This way of combining these two labels was subsequently termed dual-labeled compounds. This dual labeling allows diagnosis and therapy with one and the same molecule. Meanwhile, the surgeon becomes a real-time assistant by locating all identified malignant tissues, through which the diagnostic and therapeutic parts can act in a synergistic way [9]. This synergistic effect has been demonstrated in a study on patients using a dual-labeled antibody (NCT03699332) [10]. This additional label allows the combined use of advanced imaging techniques, such as positron emission tomography (PET) and computed tomography (CT), along with FGS, enabling precise diagnosis of primary tumors and distant metastases leading to subsequent resection of malignant tissue. FGS, which uses intraoperative fluorescence imaging, enables real-time visualization of targeted tissue through artificial staining, allowing margin delineation and thereby giving real-time feedback on tumor resection during surgery. This capability has the potential to overcome the constraints of existing intraoperative imaging techniques, enabling more precise tumor removal while minimizing damage to non-malignant tissue [11,12,13].

**Figure 2:**
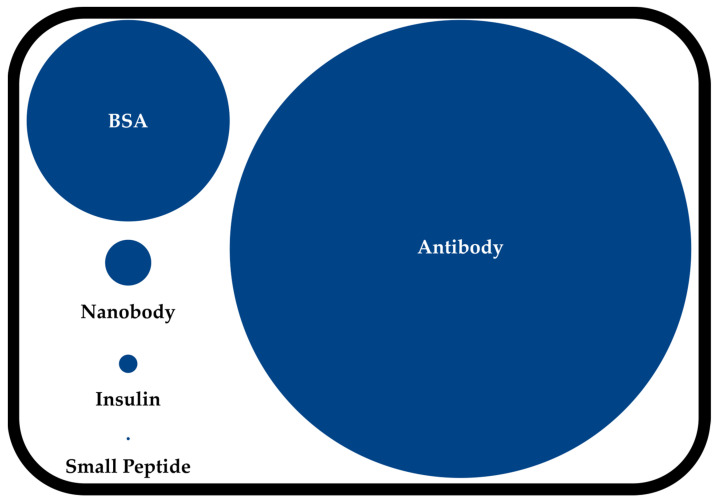
Relative comparison of sizes among an antibody, bovine serum albumin, a nanobody, insulin, and a small peptide based on the average molecular weights.

Beyond small peptides, monoclonal antibodies (mAbs) and nanobodies have established themselves as dual-labeled agents in cancer treatment [14], offering different strengths in terms of affinity, specificity, and stability in biological systems. Nanobodies represent an intermediate approach, maintaining much of the specificity of mAbs while achieving improved tissue penetration due to their reduced size. However, small peptides offer distinct advantages that render them particularly suitable for dual-labeled applications [15]. While mAbs and their derivatives can lead to prolonged circulation times and elevated background signals during imaging, the significantly reduced molecular weight of small peptides (2–4 kDa, as illustrated in Figure 2) facilitates strong tissue penetration and rapid clearance from non-target tissues and therefore less toxicity [16]. These properties are particularly beneficial for intraoperative imaging, where high contrast and minimal background are essential, most preferably at early time points after injection. Additionally, their compact size facilitates more straightforward chemical modifications and potentially more consistent dual-labeling strategies, while their shorter biological half-life can be advantageous for scheduling surgical procedures after imaging. These complementary characteristics position small peptides as valuable tools in the dual-labeling approach, particularly for applications requiring precise temporal control between diagnostic imaging.

This review focuses on small, dual-labeled peptides and peptidomimetics as targeting moieties for cancer imaging and FGS. These molecules mimic native binding motifs to achieve high binding affinities and, due to their small size, exhibit high tissue- and cell-penetrating capabilities [17] and outstanding pharmacological properties for targeting biomarkers on the surface of cancerous tissue [18,19,20]. We provide an overview of the targets addressed to date using dual-labeled small peptides and the strategies employed in their design. Additionally, we discuss the extent to which research has been translated from animal models to clinical studies.

## 2. Targets

### 2.1. Epidermal Growth Factor Receptor

The epidermal growth factor receptor (EGFR) is a crucial regulator of epithelial cell behavior and tumor activity derived from epithelial cells. EGFR belongs to the c-erbB family of receptor tyrosine kinases, which includes four homologous transmembrane growth factor receptor proteins: HER1 (c-erbB-1), HER2 (c-erbB-2), HER3 (c-erbB-3), and HER4 (c-erbB-4). EGFR, also known as HER1, was the first identified member of this family. This 170 kDa glycoprotein comprises an extracellular receptor domain, a transmembrane region, and an intracellular domain with tyrosine kinase activity [21,22]. EGFR is overexpressed in a majority of solid tumors, including those of the breast, head and neck, kidney, ovary, and colorectum as well as non-small cell lung cancer (NSCLC) [23].

Since lung cancer is the most common cancer worldwide, NSCLC is a particularly interesting target. A mutation in the EGFR gene can be used to precisely identify the biomarker [24]. This mutation can lead to increased expression by up to 89% [25], whereby the extracellular domain with its native binding pocket allows high uptake of dual-labeled compounds. In addition, internalization of the EGFR compound complex facilitates reliable localization of the radioisotope and fluorescent dye in the intended target region [26], allowing precise delineation of tumor margins.

EGFR was first targeted by a dual-labeled small peptide in 2022, where Kim et al. developed [^99m^Tc]Tc-SYPIPDT-ECG-TAMRA, incorporating SYPIPDT as the EGFR-inhibiting peptide and binding moiety. Binding affinity studies estimated the dissociation constant (K_d_) to be 76.5 ± 15.8 nM. In a mouse model bearing an NCL-H460 tumor xenograft (EGFR_WT_-positive), the tumor-to-muscle ratio (TMR) increased over time. The authors reported that compared to several other EGFR-targeting agents, [^99m^Tc]Tc-SYPIPDT-ECG-TAMRA exhibited low hepatic uptake, with high concentrations in the kidneys suggesting renal clearance [27]. In 2023, Kim et al. published a report of a modified compound, [^99m^Tc]Tc-STHHYYP-ECGK-TAMRA, designed to specifically target the EGFR_L858R_ mutation, characterized by the substitution of leucine with arginine, common in NSCLC [28]. This mutation is particularly relevant to patients who benefit most from tyrosine kinase inhibitor therapy. [^99m^Tc]Tc-STHHYYP-ECGK-TAMRA exhibited a binding affinity of 130.6 ± 29.2 nM, approximately half that of the parent compound SYPIPDT. In mouse models bearing NCL-H1650 (EGFR_L858R_-negative) and NCL-H1975 (EGFR_L858R_-positive) tumors, the TMR was twice as high as for the SYPIPDT peptide, suggesting higher specificity [29].

Kim et al. show that EGFR is a promising target for small peptides due to a significantly reduced hepatic uptake compared to antibodies, which can show values above 20 %ID/g [30]. This may result in better tolerability for the patient. However, there are also limitations, such as persistently elevated drug concentrations in blood and liver due to off-target effects as well as renal clearance, which makes it unfavorable for kidney cancer. In addition, the specific tumor accumulation of 3.48 ± 1.01 %ID/g 3 h p.i. needs to be further improved, as the dye concentration has to be stable over time for reliable assistance during FGS.

### 2.2. Folate Receptor

The folate receptor (FR) is responsible for the high-affinity uptake of folate (vitamin B9). FR is most abundant in cells essential for embryonic development, cells involved in folate uptake (such as kidney cells), and various cancerous cells, while its expression level in adult humans is overall low. There are three homologous genes encoding the FR-α, FR-β, and FR-γ isoforms, with FR-α being the most highly expressed on cancer cells [31,32].

In 2019, Kim et al. conducted the only study developing a small peptide dual-modality agent targeting FR using [^99m^Tc]Tc-Folate-ECG-ROX. In vivo imaging studies in mice bearing KB_FR+_ xenografts revealed substantial accumulation, with an increasing TMR over time (3.4 ± 0.4 %IA/g, 4.4 ± 0.7 %IA/g, and 6.6 ± 0.8 %IA/g at 1, 2, and 3 h, respectively). Additionally, competition studies with an excess of folate resulted in decreased uptake of the agent in KB tumors, further validating its FR-specific targeting. Ex vivo studies confirmed the agent’s accumulation in FR-positive tumors as well. These findings were supported by immunohistochemistry staining, which showed strong fluorescence of [^99m^Tc]Tc-Folate-ECG-ROX within KB tumor tissue. Using this imaging probe, visible tumor nodules in mice with peritoneal carcinomatosis could be removed with the support of real-time optical imaging. Immunohistochemical staining confirmed FR expression in all resected nodules [33].

Kim et al. were able to show that FR is an interesting target and that specific accumulation of dual-labeled compounds has already been demonstrated. However, the competition studies with excess folate show that follow-up compounds will probably have to use modified folate analogs to achieve a more specific binding affinity than naturally occurring folate. This could make the behavior of the compound more independent of the individual patient. Another aspect that could point folate compounds in a more specific direction is that combinatorial therapies with folate antagonists could also lead to better accumulation of the compound. By blocking the folate-metabolizing enzymes, the concentration of free folate decreases [34], which can reduce its ability to compete with the dual-labeled folate compounds. However, due to the toxicity associated with folate antagonists, such a therapeutic approach would pose new difficulties in terms of clinical translation.

### 2.3. Gastrin-Releasing Peptide Receptor

The gastrin-releasing peptide receptor (GRPR) is a 7-transmembrane G-protein-coupled receptor. Its substrate, gastrin-releasing peptide (GRP), regulates various functions of the gastrointestinal and central nervous systems through the activation of the phospholipase C signaling pathway [35,36,37]. GRPR overexpression is associated with several types of solid cancers, including gastrinomas, neuroblastomas, and GI carcinoids as well as prostate, breast, ovarian, pancreatic, colon, renal, lung, head and neck, esophageal, gastric, and uterine cancers [38].

The first team to use GRPR for dual-labeled small peptides was Zhang et al. in 2017, who reported the use of [^68^Ga]Ga-HZ220 as the first dual-modality imaging compound based on the C-terminal heptapeptide of bombesin. In vitro studies demonstrated receptor-mediated internalization, and fluorescence microscopy confirmed receptor specificity. In vivo PET/CT and fluorescence imaging of PC-3 xenografts revealed clear tumor delineation using [^68^Ga]Ga-HZ220. Biodistribution studies confirmed these findings, and fluorescence imaging showed high-intensity signals in PC-3 xenografts, corroborating PET imaging results. Subsequent autoradiography and fluorescence microscopy showed that the compound accumulated in viable regions of the xenografts, where fluorescence imaging achieved higher spatial resolution. Additionally, the authors noted an increase in renal uptake and prolonged blood circulation when the fluorophore IRDye650 was conjugated compared to the same compound without dye [39].

One year later, in 2018, Li et al. conducted the first-in-human study (NCT 02910804), treating fourteen patients with glioblastoma multiforme using [^68^Ga]Ga-IRDye800CW-BBN or [^68^Ga]Ga-BBN. All patients underwent preoperative PET imaging 30 min and 60 min p.i. and postoperative FGS 2–3 h p.i. after a single dose of 1.85 MBq per kilogram of body weight. No new neurological deficits were observed after resection. The progression-free survival (PFS) rate at 6 months was 80% [40]. Six years later, in 2024, Chen et al. conducted a study on the same compound for 52 patients with low-grade gliomas (LGG) (NCT 03407781). Group 1 (10 patients) underwent preoperative [^68^Ga]Ga-IRDye800CW-BBN PET/MRI assessment 30 min p.i. of 1.85 MBq per kilogram of body weight followed by intraoperative FGS. Group 2 (42 patients) underwent IRDye800CW-BBN FGS directly after receiving an intravenous infusion of 1.0 mg IRDye800CW-BBN. Baseline clinical characteristics revealed that intraoperative fluorescence was associated with WHO grade III gliomas (89.5%, 17/19) compared to grade II gliomas (40.0%, 8/20; *p* = 0.002). The Ki-67/MIB-1 index in the fluorescent group was higher than that in the non-fluorescent group (21.0 ± 12.5 vs. 8.3 ± 5.7; *p* = 0.001) [41]. In Group 1, preoperative PET imaging accurately predicted intraoperative fluorescence with 100% accuracy. Preoperative MRI enhancement had an accuracy of 87.2% in predicting intraoperative fluorescence. Fluorescence-guided sampling showed high sensitivity and specificity in identifying grade III tumor samples (88.6% and 88.2%, respectively) and moderate sensitivity and high specificity for grade II samples (54.7% and 88.2%, respectively). Semi-quantitative fluorescence intensity analysis showed a significantly higher signal-to-background ratio (SBR) in grade III gliomas compared to grade II gliomas and normal tissue. Patients with intraoperative fluorescence had shorter PFS and OS compared to those without fluorescence. No adverse events related to [^68^Ga]Ga-IRDye800CW-BBN were observed, and mild systemic symptoms such as nausea and sinus tachycardia were reported but resolved within 15 min. Some patients developed fever after surgery, which was attributed to either central nervous system infection or aseptic meningitis [42].

In 2022, Handula et al. developed a GRPR-targeting compound for the treatment of GRPR-positive prostate cancer. They used the inverse electron demand Diels–Alder click reaction to synthesize four variations of [^111^In]In-NeoB-DOTA-SulfoCy5, with varying linkage of the fluorophore to the well-established GRPR-targeting radiotracer NeoB [43]. Two of the tested compounds showed promising results in vivo and ex vivo in PC-3 tumor-bearing mice, with K_d_ values of 44.1 nM and 53.9 nM and TBR of 8.47 ± 0.46% ID/g and 6.90 ± 0.81% ID/g, respectively. However, uptake was also observed in the liver, lung, pancreas, and kidney [44]. Subsequently, Verhoeven et al. further investigated these compounds in 2023. They found that the compound with only one *p*ADA linker had the highest tumor-to-organ ratios (TORs) in PC-3 xenograft-bearing nude mice. The clearance rate was negatively affected by a PEG_4_ linker. A dose optimization study was conducted for the *p*ADA linker-containing compound, testing three doses (0.75, 1.25, and 1.75 nmol; 20 MBq each). The study showed that the lowest dose achieved the maximum image contrast. In addition, FGS was successfully performed with 20 MBq/0.75 nmol of the *p*ADA linker-containing compound in PC-3 and NCI-H69 xenografted mice 24 h p.i. GRPR-positive tumors could be specifically localized through preoperative SPECT/CT [37]. In 2020, Hübner et al. conducted more fundamental research focusing on how introduced fluorescent dyes influence the chemical, biological, and photophysical parameters of the final bioconjugates. They used a PESIN (PEG_3_+BBN) dimer [45] as a backbone and conjugated it with fluorophores of varying charge and hydrophobicity. The authors discovered that none of the molecular moieties affected the radiolabeling or photophysical properties. In contrast, the solubility and binding affinity were significantly influenced by the conjugated dye and correlated with its net charge. The study did not include any in vivo or ex vivo experiments [8].

These findings highlight the promise of GRPR as a target but also show that the optimization of dual-labeled GRPR-targeting compounds presents a fundamental challenge in achieving a balance between fluorescence signal intensity and radiopharmaceutical clearance dynamics. Systematic investigations show that structural modifications to enhance fluorescence properties significantly influence pharmacokinetic behavior, with dose optimization studies showing that lower doses (0.75 nmol) achieve maximum image contrast. This balance critically affects the practical time window for surgical intervention and directly impacts the ability to maintain sufficient fluorescence intensity for intraoperative guidance while ensuring adequate clearance of unbound tracer. In addition, the significant renal uptake observed with GRPR-targeting agents poses a significant challenge to their clinical translation. This characteristic biodistribution pattern has direct implications for diagnostic accuracy, particularly when imaging tumors in close proximity to the urinary tract. Clinical validation studies indicate that this physiological limitation requires careful consideration of imaging time points and protocol optimization to achieve sufficient diagnostic contrast. These findings highlight the need for integrated molecular design strategies that simultaneously optimize both imaging modalities.

### 2.4. Integrin α_V_β_3_

Integrins form a diverse superfamily of receptors, including α, β, and heterodimeric receptors, that facilitate binding between extracellular adhesion molecules and the intracellular actin cytoskeleton [46]. The α_V_β_3_ receptor is part of the Arg-Gly-Asp (RGD) motif receptors and plays a crucial role in tumor-induced angiogenesis [47], tumor metastasis [48], and even drug sensitivity [49].

This was the first target that was used for a dual-labeled small peptide: in 2010, Kimura et al. reported targeting it with a small peptide, labeled with [^64^Cu]Cu-DOTA and SulfoCy5.5. The chemical coupling was achieved using a peptide-based linker. Binding assays demonstrated preserved integrin binding affinity despite the addition of imaging labels. In vivo studies demonstrated specific tumor targeting and a good correlation between NIRF and PET imaging. Biodistribution studies demonstrated tumor-specific accumulation, with TBR of 3.24 ± 0.87 %ID/g, 5.64 ± 0.83 %ID/g, and 4.57 ± 0.70%ID/g at 1, 4, and 24 h p.i. Metabolic stability analysis indicated that the probe metabolizes in tumor tissue, while stability was observed in blood and serum [50].

Two years later, in 2012, Liu et al. published a study on a small peptide labeled with a sarcophagine cage for radioactive labeling and SulfoCy5.5 as a fluorophore. The authors reported tumor uptake of 6.41 ± 0.28 %ID/g, 6.51 ± 1.45 %ID/g, and 5.92 ± 1.57 %ID/g at 1 h, 4 h, and 20 h p.i., respectively [51]. Between 2011 and 2014, the authors also employed a dual-labeling strategy using a BODIPY dye that can be directly labeled with [^18^F]F. They reported specific binding to the integrin αvβ3 receptor and demonstrated tumor uptake both in vivo and ex vivo using a U87MG mouse model. Furthermore, a strong signal was observed in the liver and kidney [52,53,54].

Another integrin-targeting compound based on the same RGD peptide was reported in 2018 by Sun et al.; it was tested in vivo in a U87MG xenograft model. The authors used an innovative fluorophore that absorbs light in the second near-infrared region (NIR-II), providing advantages such as better tissue penetration, minimal tissue absorption, and low autofluorescence [55,56]. In the same study, Sun et al. demonstrated base-catalyzed thiol-yne chemistry, which offers a straightforward approach to develop dual-modality probes. The TBR reached 4.77 ± 0.26 %ID/g at 12 h p.i. They also reported renal and hepatobiliary excretion, with signals in the kidney and liver at 60 h p.i. [56].

Analysis of integrin-targeting agents reveals several significant technological advances in molecular design and imaging capability. The implementation of NIR-II fluorophores represents a significant advancement in imaging depth and resolution, providing superior tissue penetration compared to conventional NIR-I fluorophores. This improvement is due to reduced photon scattering and minimal tissue absorption in the NIR-II window, resulting in improved signal-to-noise ratios and reduced autofluorescence [57]. However, the efficacy of fluorophore behavior is intrinsically linked to differential metabolic profiles, where retention of parent compounds in the circulation coupled with tumor-specific metabolic processing facilitates enhanced TBR. These findings highlight the critical importance of rationally designed molecular architectures. To facilitate the efficient design of such compounds, base-catalyzed thiol-yne click chemistry has emerged as a valuable synthetic strategy, enabling site-specific conjugation through selective thiol-alkyne reactions under mild conditions. This methodology is of particular importance for dual-labeled compounds, where precise control over the conjugation of two different imaging moieties to the targeting vector is essential.

### 2.5. Matrix Metalloproteinase

There are currently 23 known human matrix metalloproteinases (MMPs), forming a family of orthologous peptidases involved in various aspects of extracellular matrix remodeling [58]. MMPs are major players in angiogenesis and vasculogenesis, processes that are often highly upregulated during malignant cell growth [59]. Additionally, studies have shown that MMPs contribute to the formation of pre-metastatic niches, facilitating the escape of cancer cells from the primary tumor and their metastasis to distant sites [60].

In 2011, Azhdarinia et al. were among the first to demonstrate the applied use of a dual-labeled tracer targeting MMP-2/9, which is involved in angiogenesis in bone growth plates. The small inhibitor peptide KKAHWGFTLD was labeled with [^68^Ga]Ga-DOTA and IRDye800CW [61]. Their compound was tested in vivo in a mouse model of heterotopic ossification, showing promising results in PET/NIR imaging and observed clearance through the kidneys [62].

Nearly a decade later, a highly innovative approach was published by Kasten et al. in 2020. They developed a novel substrate-binding peptide targeting the collagenase MMP-14, which activates the fluorophore IRDye800. This fluorophore is quenched by IR QC-1, allowing detection of the NIRF signal only upon MMP-14 processing. This approach improves probe specificity and imaging contrast, as MMP-14 expression is negligible in healthy tissue. The probe was tested in vivo in mice with three different mutated orthotopic patient-derived xenograft glioma tumors. The study demonstrated the probe’s specific binding to glioma cancer cells, with high-NIRF-signal TBRs in the resected brain samples but relatively low PET signals [63]. Nevertheless, the approach described by Kasten et al. can only be applied to selected targets, as it relies on proteolytic activity. If so, this approach could lead to fewer side effects and preferably less systemic toxicity.

### 2.6. Neurotensin Receptor

The neurotensin receptor 1 (NTSR1) is a G protein-coupled receptor (GPCR) that belongs to the ghrelin receptor family. It is activated by the endogenous 13-amino acid peptide agonist neurotensin (NTS) and the NTS-related hexapeptide neuromedin N. The C-terminal hexapeptide portion of NTS, NTS^8–13^, represents the binding epitope for NTSR1 activation [64]. NTSR1 is implicated in various pathological conditions, including psychostimulant addiction, schizophrenia, Parkinson’s disease, neurodegenerative diseases, intestinal inflammation, obesity, metabolic disorders, and cardiovascular diseases. Dysregulated NTSR1 signaling also enhances the proliferation, survival, and metastatic spreading of many cancers, often associated with a worse prognosis for patients [65,66].

To address this issue, Deng et al. developed [^64^Cu]Cu-DOTA-NT-SulfoCy5.5 in 2015. The compound targets the NTR1 receptor in HT-29 tumor-bearing mice through NTS^8–13^. It demonstrated high stability in vitro and specific accumulation in NTR1-expressing tumor lesions (1.91 ± 0.22 %ID/g and 1.79 ± 0 0.16 %ID/g at 1 and 4 h p.i., respectively), confirmed by PET and fluorescence imaging. Receptor specificity was validated through blocking experiments (0.42 ± 0.05 %ID/g). The IC_50_ was determined to be in the nanomolar range (0.65 ± 0.35 nM), while biodistribution studies showed predominant renal clearance and high TMR (17.44 ± 3.25 %ID/g at 4 h p.i., respectively), indicating the potential as a PET tracer. However, moderate tumor uptake and variations in liver uptake were observed in vivo, suggesting the need for further optimization. Proof-of-principle FGS successfully identified and removed tumors under fluorescent guidance [67].

However, the presence of low accumulation in the tumor and about twice the concentration in the liver suggests that much development is needed to consider NTSR1 as a promising biomarker for dual-labeled compounds.

### 2.7. Prostate-Specific Membrane Antigen

**Figure 3:**
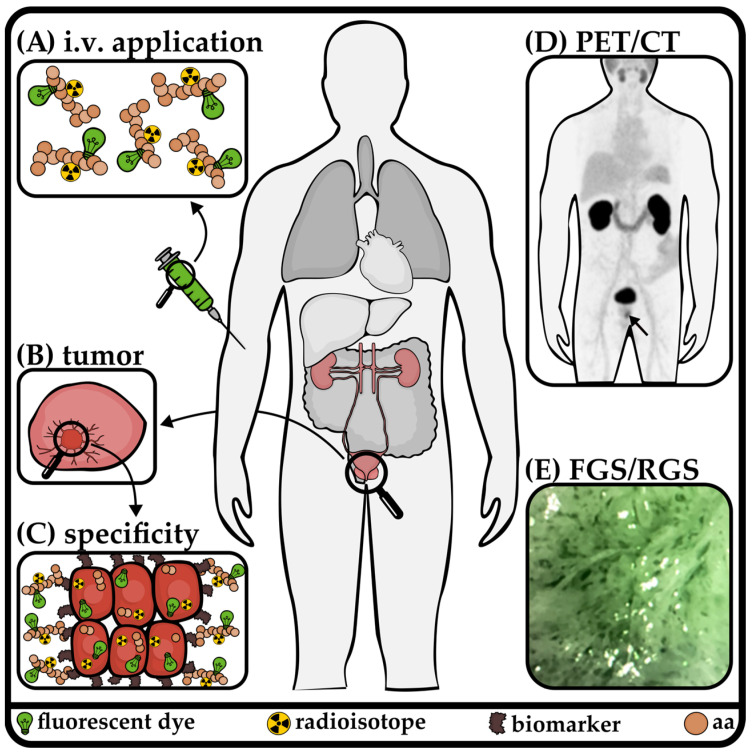
Sketch from application to therapy, demonstrated on prostate cancer. A small peptide consisting of amino acids (aa) is dual-labeled with fluorescent dye (illustrated as a green bulb) and a radioisotope (radioactive symbol). After application (**A**), the compound accumulates specifically in the targeted prostate tumor (**B**) through biomarkers on the cell surface, which also lead to internalization (**C**). Subsequently, diagnostic PET/CT [75] (**D**) and therapeutic FGS/RGS [75] (**E**) can be performed.

Prostate-specific membrane antigen (PSMA) is a transmembrane protein expressed by prostate epithelial cells. It is also known as folate hydrolase 1 (FOLH1), glutamate carboxypeptidase II (GCPII), or N-acetyl-α-linked acidic dipeptidase I. PSMA is highly overexpressed in more than 85% of patients with prostate cancer [68]. To illustrate the path of a dual-labeled peptide from administration to diagnostic imaging and surgical guidance, we have used a PSMA-targeting dual-labeled compound as an example in Figure 3. Although the function of PSMA in prostate cancer remains unclear, higher PSMA expression is associated with decreased survival. Beyond the prostate, PSMA is expressed in other tissues such as renal proximal tubules, small bowel, salivary and lacrimal glands, neovasculature, and many solid tumors [69]. PSMA inhibitors are among the pioneering dual-modalities for the treatment and diagnosis of other tumor types, including renal cell carcinoma [70]. One key characteristic of PSMA-targeting agents is their internalization through receptor-mediated endocytosis [71,72,73,74].

In 2018, Baranski et al. demonstrated that conjugating fluorescent dyes to a PSMA-11-derived precursor does not affect PSMA binding affinity. The labeled compounds showed improved cell surface binding and internalization in PSMA-positive LNCaP cells, as demonstrated by confocal microscopy. In vivo characterization showed that the dual-labeled conjugates had higher tumor uptake compared to the reference, [^68^Ga]Ga-PSMA-11. The pharmacokinetic properties were preserved, and there was specific uptake in PSMA-expressing LNCaP tumor xenografts. Organ distribution studies showed stable and high tumor uptake over time with reduced non-specific uptake. Small-animal PET studies revealed tumor visualization and rapid clearance from background organs. Proof-of-concept studies demonstrated PSMA-specific fluorescence in tumor tissue ex vivo. Furthermore, FGS has been successfully conducted in pigs as a large-animal model, demonstrating the potential for clinical translation [76]. Further studies focused on the linker design of this first-generation dual-labeled compound EuK-HBED-CC-IRDye800CW. Improvements were validated through in vitro studies, confirming high PSMA affinity in the nanomolar range. The insertion of histidine–glutamic acid (HE) or tryptophan–glutamic acid (WE) linkers did not affect the specific binding and internalization properties compared to the core structure. Biodistribution studies indicated improved tumor uptake and reduced background organ enrichment for [^68^Ga]Ga-EuK-(HE)_3_-HBED-CC-IRDye800CW compared to other motifs or positioning variations. The compound performed better than established PSMA tracers, for example, reducing spleen uptake from 38.12 ± 14.62 %ID/g to 3.47 ± 1.39 %ID/g. Small-animal PET imaging confirmed strong tumor uptake and rapid clearance from non-malignant tissue, demonstrating high PSMA specificity. Fluorescence imaging showed excellent tumor visualization and confirmed the renal excretion pathway and reduced spleen uptake [77]. The second-generation dual-labeled PSMA-914 compound was tested in the same year for the first preoperative PET/CT imaging and FGS in a patient with high-risk prostate cancer (Gleason score 9 [4 + 5] and initial PSA level 7 ng/mL). PET/CT imaging was performed at 1 h p.i. and showed strong uptake in the primary tumor. The day after, during surgery, PSMA-914 facilitated precise tumor visualization and delineation. Ex situ fluorescence detection confirmed the tumor-specific enrichment of PSMA-914 and its high contrast to surrounding healthy tissues, but further clinical studies are needed for validation [75]. Building on these promising results, Eder et al. published another dual-labeled PSMA ligand in 2022, based on the clinically evaluated PSMA-617 precursor. In vitro evaluation with PSMA-expressing LNCaP cells showed that the introduction of IRDye800CW to PSMA-617 via the lysine branch maintained low nanomolar PSMA-binding affinity (13.22 ± 5.25 nM). The optimal dye installation was determined at the ε-position of the lysine side chain, with PSMA-927 exhibiting the highest specific cell surface binding (25.51 ± 9.73 %AR/10^5^ cells) and internalization rates (27.64 ± 12.80 %AR/10^5^ cells) among the library candidates. Fluorescence microscopy confirmed PSMA-specific binding and internalization of PSMA-927 without causing significant cytotoxic effects in cell proliferation studies. Subsequent in vivo studies using [^68^Ga]Ga-PSMA-927 demonstrated high tumor accumulation in a PSMA-positive xenograft mouse model, comparable to that of [^68^Ga]Ga-PSMA-617. PET/MRI and fluorescence imaging further confirmed the compound’s high PSMA specificity and tumor uptake, with rapid clearance from non-malignant tissues via the renal pathway [78].

Also in 2018, Kommidi et al. published a report of a dual-labeled PSMA inhibitor based on the same EuK-binding motif. The ligand was labeled with ^18^F[F] and a cyanine derivative, resulting in a compound with a binding affinity in the nanomolar range (6.74 ± 1.33 nM) for PSMA-expressing PC3-PIP cells. The ligand also showed specific binding and internalization. PET/CT analysis in mice revealed clear uptake of [^18^F]F-4 in PSMA-expressing tumor tissue, with minimal uptake in background tissues. Ex vivo γ-scintillation further confirmed the biodistribution of [^18^F]F-4, with 175-fold higher accumulation in PSMA-expressing tissue compared to control. Fluorescence imaging confirmed the PET findings, demonstrating a 5.2-fold increase in fluorescence intensity in PSMA-expressing tumor tissue compared to control tissue [79]. In 2021, Aras et al. reported the first-in-human study of [^18^F]F-4, now [^18^F]F-BF3-Cy3-ACUPA, as a potential PET imaging agent for prostate cancer and other PSMA-expressing tumors. The study included ten patients (66 ± 7 years) with suspected prostate cancer (9) and colorectal cancer (1). The compound was well-tolerated with no reported adverse effects. PET imaging revealed favorable biodistribution patterns, with notable accumulation in PSMA-expressing tumors. Radiation dosimetry analysis indicated acceptable levels of radiation exposure, with high uptake observed in the salivary glands, kidneys, and lacrimal glands. Comparison to other PSMA-targeted tracers demonstrated similar or lower radiation doses. PET imaging also revealed high TBR in PSMA-expressing lesions and lymph nodes. All patients underwent preoperative PET imaging and received an average dose of 6.5 ± 3.2 mCi of [^18^F]F-BF3-Cy3-ACUPA. On average, 73 ± 27 min passed between injection and image acquisition. Intraoperative fluorescence imaging facilitated the identification of PSMA-positive lesions and lymph nodes, aiding in surgical resection. The presence of PSMA expression in resected tissue was confirmed by post-surgical histopathology and multi-photon microscopy [80]. In the same year, Aras et al. further reported a study about a 74-year-old patient with elevated PSA levels (8 ng/mL) undergoing pre-biopsy PET imaging using both [^68^Ga]Ga-PSMA and [^18^F]F-BF3-Cy3-ACUPA probes. Both probes showed increased uptake in the right peripheral zone of the prostate, indicating PSMA-positive cancer. [^18^F]F-BF3-Cy3-ACUPA was co-injected with non-radioactive Cy7-ACUPA. Despite the known differences in chemical characteristics of Cy3 and Cy7, the authors also reported similar in vivo pharmacokinetic profiles and targeting properties regarding PSMA. Fusion biopsy confirmed Gleason 8 (4 + 4) prostate cancer. Ex vivo fluorescence microscopy of targeted biopsy samples demonstrated specific binding of both Cy3 and Cy7 ACUPA fluorophores to PSMA-positive tumor foci. Despite differences in tissue penetration, both fluorophores showed similar pharmacokinetic profiles and specific targeting to PSMA [81].

In 2019, Schottelius et al. developed a dual-labeled PSMA inhibitor (PSMA-I&F) based on the previously established tracer PSMA-I&T [82]. They demonstrated that the linker extension and conjugation with the fluorophore Sulfo-Cy5 dye slightly increased the lipophilicity of the compound. This led to stronger non-specific binding to human serum albumin (from 83% to 96.5%) without affecting PSMA affinity (IC_50_ 10.5 ± 2.1 nM to 9.6 ± 1.7 nM). The authors observed internalization into PSMA-expressing LNCaP cells and rapid shuttling into lysosomes. In vivo biodistribution studies revealed rapid background clearance and high, sustained renal uptake of [^177^Lu]Lu-PSMA-I&F. Similarly, [^68^Ga]Ga-PSMA-I&F exhibited comparable distribution patterns with increased renal uptake. Small-animal PET imaging confirmed high-contrast imaging of PSMA-expressing tumors with rapid background clearance. Fluorescence imaging of cryosections closely correlated with PET imaging results, demonstrating the accumulation of PSMA-I&F in tumors, kidneys, and salivary glands. PSMA immunohistochemistry and fluorescence microscopy showed specific tracer accumulation in tumor and kidney tissues. Proof-of-concept FGS of PSMA-expressing prostate carcinoma tissue in vivo was also performed in a mouse model carrying LNCaP xenograft tumors [83]. In the following year, Hensbergen et al. investigated the characteristics of different spacers between the EuK-Cy5-mas3 moiety to enhance pharmacokinetics and pharmacodynamics. They found that the functionalization site affected lipophilicity and that the degree of plasma protein binding (PPB) was correlated with lipophilicity. Tracers with higher lipophilicity showed higher PPB. In vitro imaging showed variations in cellular binding among spacer localization. EuK-(SO_3_)-Cy5-mas3 exhibited the highest binding affinity (19.2 ± 5.8 nM) to PSMA-overexpressing PC346C cells. Blocking studies resulted in significant reductions in tracer uptake, indicating specific binding. Tracers with sulfonate moieties revealed higher PSMA affinity, suggesting favorable interactions with the target [84].

Since 2021, Derk et al. have been investigating a new application for dual-labeled small peptides in photodynamic therapy. They used IRDye700DX as a photosensitizer-based compound instead of a fluorophore. Multiple inhibitors were designed with different combinations of linkers, chelators, and IRDye700DX. In vitro assays demonstrated specific binding (K_d_ 6.9 ± 3.5 nM) and internalization of all inhibitors in artificially PSMA-expressing LS174T cells, with certain inhibitors presenting higher uptake. In vivo studies demonstrated that the inhibitors showed PSMA-specific tumor uptake, with N064 ([^111^In]In-EuK-D-Phe-Glu-Glu-DOTAGA-IRDye700DX) presenting the best results. Multi-modal imaging using SPECT/CT and fluorescence imaging successfully visualized PSMA-positive tumors in mice and aided tumor resection. Human prostate cancer biopsies were incubated with 0.08 nmol (molar activity 26.3 MBq/nmol) of either [^111^In]In-PSMA-N064 or [^111^In]In-PSMA-N140 and showed preferential accumulation of dual-labeled inhibitors in tumor tissue compared to normal tissue, as confirmed by fluorescence and autoradiography [85]. In the following year, Derks et al. continued to refine the linker design, resulting in three new compounds. They combined their N064 with the high-affinity ligands PSMA-1007 and PSMA-617 and substituted one glutamate acid with two additional lysine residues in the linker region to further functionalize those side chains for higher tumor uptake. Their best candidate, N064, achieved binding affinities in the nanomolar range (K_d_ = 6.9 nM), internalization rate of 4.6 ± 0.31 % and tumor uptake rate of 15.1 ± 0.8 %ID/g 2 h p.i. in a LS174T colon carcinoma xenograft mouse model, resulting in reduced cell viability by targeted photodynamic therapy. Since the LS174T cell line is stably transfected with DNA encoding for human PSMA, the PSMA occurrence is supposed to be artificially high [86]. In the same year, Derks et al. reported the use of a SPAAC chemistry-based approach and compared it with the more commonly used NHS approach for the rapid development of new dual-labeled compounds. They found no significant differences between the resulting ligands. The authors concluded that the use of click chemistry could be advantageous in the development of new compounds due to its standardized synthesis and mild reaction conditions [87].

A different approach was pursued in 2021 by Kim et al. investigating the GRFLTGGTGRLLRIS [88] peptide as a PSMA binding motif for the first time beyond the most abundant used EuK binding motif. In vitro binding assays showed high affinity (K_d_ 9.5 ± 1.3 nM), and confocal microscopy confirmed strong fluorescence activity for PSMA-positive LNCaP cells. In vivo gamma camera imaging in mice demonstrated significant accumulation in PSMA-positive LNCaP tumors over time, with increasing TMR. Minimal accumulation was observed in the control group. Biodistribution studies and immunohistochemistry staining also confirmed the presence of PSMA-specific fluorescence in LNCaP tumor tissue. Additionally, real-time optical imaging enabled successful removal of visible tumor nodules in mice with peritoneal carcinomatosis [89].

Another alternative PSMA-binding moiety based on ODAP–urea was published by Duan et al. in 2020 [90]. Based on this, Li et al. constructed in 2022 four dual-labeled ligands based on the ODAP–urea PSMA-targeting moiety and varying lengths of carboxylate-modified linkers. The ligands exhibited absorption spectra peaking around 808 nm and demonstrated suitable optical properties for in vivo NIR imaging with good photostability. The in vitro evaluation revealed that all ligands underwent potent PSMA binding, with similar inhibition constants (K*_i_* = 2.09 ± 1.71 nM to 4.15 ± 2.20 nM). The ligands’ hydrophilicity increased with the number of carboxylate linkers, while their cellular uptake was highest with [^68^Ga]Ga-P3 (n_carboxylate_ = 4). In vivo PET/CT imaging in a PSMA-expressing tumor model demonstrated that [^68^Ga]Ga-P3 has superior pharmacokinetic properties. Biodistribution studies confirmed the specific tumor targeting of [^68^Ga]Ga-P3 in 22Rv1 xenograft model. The TMR and TLR were 10.24 ± 4.42 %ID/g and 3.99 ± 0.87 %ID/g at 1 h p.i., NIR-I imaging of P3 in 22Rv1 tumor-bearing mice showed selective tumor accumulation with good contrast, both in vivo and ex vivo. Toxicity studies in mice demonstrated no apparent acute toxicity or pathological abnormalities, suggesting a good safety profile [91].

The most recent study on a dual-labeled small peptide targeting PSMA was published in 2023 by Fu et al. reporting the use of the novel compound NYM016 with the near-infrared fluorescent moiety Cyanine-7 and the chelating group NOTA. In vitro experiments confirmed NYM016’s high affinity for PSMA in LNCaP cells (K_d_ 0.335 ± 0.125 nM). In a mouse model, NYM016 demonstrated specific accumulation in PSMA-expressing LNCaP cells, as evidenced by NIRF imaging. NIRF imaging was used to aid in the surgical resection of PCa tumors in mice. Persistent fluorescence aggregation was observed in tumors and kidneys up to 24 h p.i., indicating stable and specific targeting of NYM016 to PSMA-positive tumor sites. Furthermore, NYM016 was safely administered to two patients with recurrent PC and prostatitis (NCT05623878). Both patients received 1 mg/74 MBq of [^68^Ga]Ga–NYM016 i.v., PET/CT was performed 1 h p.i. and prostatectomy 2 h p.i. [^68^Ga]Ga–NYM016 aggregated in the excised lesion, confirming its ability to target PSMA in vivo. PET/CT imaging using [^68^Ga]Ga-NYM016 successfully identified recurrent lesions and metastases in a patient with prostate cancer. The compound showed weak or no retention in the prostate of a patient with prostatitis [92].

## 3. Summary

As presented in Table 1, there are substantial variations in the extent to which biomarkers were prioritized in the development of dual-labeled small peptides. PSMA-targeting compounds have demonstrated the most substantial progress in clinical translation, which can be attributed to PSMA’s well-established role as one of the most extensively studied targets in radiopharmaceutical development. This strong foundation in nuclear medicine, coupled with proven clinical success of radioactive PSMA inhibitors, has facilitated rapid advancement in dual-labeled approaches. The evolution of these compounds, particularly through optimization of linker designs and coupling strategies, has led to improved pharmacokinetic profiles with rapid clearance from non-target tissues while maintaining high tumor uptake. Notable achievements include the clinical validation of PSMA-914, which showed excellent tumor visualization capabilities and specific binding in surgical settings and the preclinical development of PSMA-I&F.

GRPR-targeting agents have shown significant clinical success, particularly in neuro-oncology. Clinical trials have demonstrated high sensitivity and specificity in identifying different grades of gliomas, thus establishing GRPR as a valuable target for image-guided surgery. Recent developments in GRPR-targeting compounds, including novel spacer designs and photosensitizer-based approaches, indicate expanding therapeutic possibilities beyond traditional applications.

EGFR-targeting compounds represent a promising avenue, particularly for NSCLC treatment where EGFR mutations are prevalent. While encouraging results have been observed thus far, ongoing research endeavors are directed towards optimizing tumor accumulation and reducing off-target effects. The development of compounds that are specifically designed to target EGFR mutations underscores the potential for personalized medicine approaches in this field.

The remaining molecular targets have also demonstrated distinct advantages and potential applications. Folate receptor targeting has exhibited promising specificity; however, the development of more selective analogs may be necessary to overcome competition with endogenous folate. Integrin α_V_β_3_ targeting has exhibited a pioneering approach with the use of a NIRII fluorophore, which has significantly improved imaging capabilities. Nevertheless, the metabolic processing of the compound has proven challenging. MMP-targeting compounds have introduced innovative activation mechanisms through quenched fluorophores, offering improved specificity through targeted activation and the potential to achieve better safety profiles in the future. The neurotensin receptor approach has lacked critical pharmacological characteristics, and the absence of further research publications suggests that it may not be promising enough.

The path to clinical adoption, however, is complicated by several challenges. A significant hurdle lies in the substantial investment required for specialized medical infrastructure, particularly advanced surgical systems such as robotic-assisted surgical platforms, which are essential for optimal utilization of FGS. These systems represent considerable financial commitments for healthcare institutions, potentially limiting accessibility of technology. The regulatory pathway for dual-labeled compounds presents another layer of complexity. To ensure the widespread adoption of these technologies, it is essential to demonstrate their significant clinical benefit compared to well-established single-labeled modalities. Large-scale patient studies are necessary to substantiate this benefit and justify widespread adoption. However, conducting such studies demands extensive investment, even before clear evidence of clinical advantage is available. While current clinical data show promise, they are primarily derived from smaller studies or single-center experiences. This underscores the necessity of more extensive multicenter trials to ascertain the cost-effectiveness and clinical benefit of these dual-modality approaches in comparison to current standard-of-care imaging techniques.

**Table 1:** Summary of all dual-labeled small peptide compounds.

Author	Biomarker	Cancer	Binding Motive	Fluorophore	Chelator	Radionuclide	Patients	Reference
Kim et al. (2022) [27]	EGFR	NSCLCL	SYPIPDT	TAMRA	ECG	Tc-99m	-	-
Kim et al. (2023) [29]	EGFRL858R	NSCLCL	STHHYYP	TAMRA	ECGK	Tc-99m	-	-
Kim et al. (2019) [33]	FR	FR+	Folate	ROX	ECG	Tc-99m	-	-
Li et al. (2018) [40]	GRPR	GBM	BBN	IRDye800CW	NOTA	Ga-68	14	NCT 02910804
Chen et al. (2024) [42]	GRPR	LGG	BBN	IRDye800CW	NOTA	Ga-68	39	NCT 03407781
Hübner et al. (2020) [8]	GRPR	-	PESIN-dimer	Diverse	NODA	Ga-68	-	-
Handula et al. (2022) [44]	GRPR	PC (GRPR+)	NeoB	Sulfo-Cyanine 5	DOTA	In-111	-	-
Zhang et al. (2017) [39]	GRPR	PC (GRPR+)	HZ220	IRDye650	DOTA	Ga-68	-	-
Verhoeven et al. (2023) [37]	GRPR	PC (GRPR+)	NeoB	Sulfo-Cyanine 5	DOTA	In-111	-	-
Liu et al. (2013) [51,52,53,54]	Integrin αvβ3	GBM	RGD	BODIPY R6G	-	F-18	-	-
Sun et al. (2018) [56]	Integrin αvβ3	GBM	RGD	CH1055	NOTA	Ga-68	-	-
Azhdarinia et al. (2011) [62]	MMP-9	HO	KKAHWGFTLD	IRDye800CW	DOTA	Ga-68	-	-
Kasten et al. (2020) [63]	MMP-14	Glioma	HWKHLHNTKTFL	IRDye800CW	NOTA	Ga-68	-	-
Deng et al. (2015) [67]	NTR1	Colon (NTR+)	pELTENKPRRPTIL	Sulfo-Cyanine 5	DOTA	Cu-64	-	-
Baranski et al. (2018) [76]	PSMA	PC (PSMA+)	EuK	IRDye800CW	HBED	Ga-68	-	-
Kommidi et al. (2018) [79]	PSMA	PC (PSMA+)	EuK	Sulfo-Cyanine 3	BF3	F-18	-	-
Schottelius et al. (2019) [83]	PSMA	PC (PSMA+)	EuK	Sulfo-Cyanine 5	DOTAGA	Ga-68/Lu-177	-	-
Hensbergen et al. (2020) [84]	PSMA	PC (PSMA+)	EuK	IRDye800CW	MAS3	Tc-99m	-	-
Kim et al. (2020) [89]	PSMA	PC (PSMA+)	GRFLTGGTGRLLRIS	ROX	ECG	Tc-99m	-	-
Eder et al. (2021) [77]	PSMA	PC (PSMA+)	EuK	IRDye800CW	HBED	Ga-68	-	-
Eder et al. (2021) [75]	PSMA	PC (PSMA+)	EuK	IRDye800CW	HBED	Ga-68	1	-
Aras et al. (2021) [80,81]	PSMA	PC (PSMA+)	EuK	Sulfo-Cyanine 3	BF3	F-18	10	-
Derks et al. (2021) [85]	PSMA	PC (PSMA+)	EuK	IRDye700DX	DOTA/DOTAGA	In-111	-	-
Derks et al. (2021)-2 [86]	PSMA	PC (PSMA+)	EuK	IRDye800DX	DOTA	Tc-99m/In-111	-	-
Derks et al. (2022) [87]	PSMA	PC (PSMA+)	EuK	IRDye700DX	DOTA	In-111	-	-
Eder et al. (2022) [78]	PSMA	PC (PSMA+)	EuK	IRDye800CW	DOTA	Ga-68	-	-
Li et al. (2022) [91]	PSMA	PC (PSMA+)	(ODAP)-Urea	ICG	DOTA	Ga-68	-	-
Fu et al. 2023) [92]	PSMA	PC (PSMA+)	NYM016	Sulfo-Cyanine 7	NOTA	Ga-68	2	NCT05623878

## 4. Outlook

Since the basis for dual-labeled cancer theranostics using small peptides was established approximately 20 years ago, significant advancements have been achieved in its further development. The frequency of new publications in this field has steadily increased. Intensive research has been conducted on the fundamental components, including chelators, linkers, fluorophores, and peptides [93,94,95], which has continuously improved the pharmacological properties of the compounds. However, it is equally crucial to emphasize the need for a comprehensive understanding and addressing of the impact of receptor dynamics, particularly ectodomain shedding. A significant number of targeted receptors have been reported to undergo cleavage and release into circulation, which may potentially affect the quality of imaging and the efficacy of therapeutic treatment. While the current clinical outcomes suggest that this phenomenon could lead to a reduction in the effectiveness of dual-labeled compounds [96], further data are required to determine which targets could benefit most from an adapted design. Technical advances have enabled the identification of increasingly precise biomarkers [97], whereby the constantly evolving phage displays have led to a high throughput of binder design [98]. Additionally, AI-supported binder design represents a promising avenue for further progress [99,100], and the technique of FGS is rapidly evolving [101,102]. Recent advances in AI-driven protein structure prediction and design could significantly enhance the development of more specific targeting peptides with improved binding affinities [99]. By optimizing binding specificity through computational modeling of peptide–target interactions, AI approaches may enable the development of less off-targeting compounds, potentially reducing systemic toxicity [103]. Nevertheless, the advent of target-specific modeling of small-peptide binders may in the future allow for the design of small-peptide binders against novel targets that were not addressed by small peptides yet [104]. While significant progress has been made in the development of dual-labeled peptides for targets such as PSMA, EGFR, and GRPR, there are several promising cancer biomarkers that have yet to be explored through this approach. One such example is HER2, which is overexpressed in approximately 20–30% of breast cancers and has proven successful as a target for small-peptide compounds [105]. Beyond the identification of new binders, AI models trained on extensive biodistribution data could also help optimize the pharmacokinetic properties of dual-labeled compounds in the future, potentially leading to reduced off-target accumulation [106].

This field of research has been successfully translated from the laboratory to the early clinic, where it has achieved great success in individualized cancer therapy for patients [40,42,75,80,92]. Nevertheless, this field, while promising, remains in its early developmental stages. Although the scaling up of the production of dual-labeled compounds may appear challenging at first glance, many of the necessary infrastructure components are already in place. The established pipeline for radiopharmaceutical kit production provides a robust foundation that can be adapted for dual-labeled agents. While these compounds necessitate specific protocols and modified precursors to accommodate the additional fluorescent label, the fundamental principles and quality control processes remain analogous to existing radiopharmaceutical production methods. The radiochemistry facilities, hot cells, and automated synthesis modules currently employed for single-labeled compounds can be readily adapted for dual-labeled agent production. The regulatory framework and good manufacturing practice guidelines established for radiopharmaceuticals provide a clear pathway for clinical translation. While the initial setup costs may be higher due to the need for additional quality control measures for the fluorescent component, the long-term scalability should be achievable within existing radiopharmaceutical infrastructure. Consequently, while the production costs of dual-labeled agents may potentially exceed those of single-labeled agents, they should not constitute an insurmountable barrier to clinical implementation. To ascertain the true added value of promising active substances compared to current therapeutic practices, it is essential to conduct studies on larger patient cohorts. Another crucial factor will be determining the appropriate dose for patients, which has thus far played a relatively subordinate role. For this approach to transition from a niche application to more widespread use, it is essential to prioritize its practicality within a clinical setting. While the results thus far are encouraging, it is imperative to place greater emphasis on the distinctive characteristics of the fluorophore as a label. The objective is to differentiate the targeted tissue from its surrounding environment in a manner that allows a clear distinction between healthy and malignant tissue, whether by an operator or even by a robot. This necessitates close collaboration with medical technology, as this is the only means of facilitating genuine clinical translation. Numerous future approaches are anticipated to advance patient-centered therapy. In order to ensure the continuity of this progress and to accelerate the clinical translation of dual-labeled compounds, there are several key initiatives that must be prioritized. First, the establishment of standardized protocols for the development, characterization, and quality control of dual-labeled compounds would result in a streamlined regulatory approval process and facilitate multicenter clinical trials. Second, the fostering of closer collaboration among academic institutions, medical centers, and industry partners through dedicated research networks could expedite the transition from preclinical studies to clinical implementation. The establishment of such networks would facilitate the sharing of resources, expertise, and patient populations, thereby enabling more efficient clinical trials. The development of standardized imaging protocols and analysis methods would also allow the comparison of results across different centers and studies, contributing to the advancement of precision oncology. This collaborative approach, coupled with ongoing technological innovation, has the potential to establish dual-labeled compounds as a standard tool in precision oncology.

## Data Availability

No new data were created or analyzed in this study. Data sharing is not applicable to this article.
